# Single-atom-layer traps in a solid electrolyte for lithium batteries

**DOI:** 10.1038/s41467-020-15544-x

**Published:** 2020-04-14

**Authors:** Feng Zhu, Md Shafiqul Islam, Lin Zhou, Zhenqi Gu, Ting Liu, Xinchao Wang, Jun Luo, Ce-Wen Nan, Yifei Mo, Cheng Ma

**Affiliations:** 1 Division of Nanomaterials & Chemistry, Hefei National Laboratory for Physical Sciences at the Microscale, CAS Key Laboratory of Materials for Energy Conversion, Department of Materials Science and Engineering, University of Science and Technology of China, Hefei, Anhui 230026 China; 2 Department of Materials Science and Engineering, University of Maryland, College Park, MD 20742 USA; 3 Ames Laboratory, U.S. Department of Energy, Ames, IA 50011 USA; 4 School of Materials Science and Engineering, State Key Laboratory of New Ceramics and Fine Processing, Tsinghua University, Beijing, 100084 China; 5 Center for Electron Microscopy and Tianjin Key Lab of Advanced Functional Porous Materials, Institute for New Energy Materials & Low-Carbon Technologies, School of Materials Science and Engineering, Tianjin University of Technology, Tianjin, 300384 China

**Keywords:** Materials for energy and catalysis, Batteries

## Abstract

In order to fully understand the lithium-ion transport mechanism in solid electrolytes for batteries, not only the periodic lattice but also the non-periodic features that disrupt the ideal periodicity must be comprehensively studied. At present only a limited number of non-periodic features such as point defects and grain boundaries are considered in mechanistic studies. Here, we discover an additional type of non-periodic feature that significantly influences ionic transport; this feature is termed a “single-atom-layer trap” (SALT). In a prototype solid electrolyte Li_0.33_La_0.56_TiO_3_, the single-atom-layer defects that form closed loops, i.e., SALTs, are found ubiquitous by atomic-resolution electron microscopy. According to ab initio calculations, these defect loops prevent large volumes of materials from participating in ionic transport, and thus severely degrade the total conductivity. This discovery points out the urgency of thoroughly investigating different types of non-periodic features, and motivates similar studies for other solid electrolytes.

## Abstract (web summary)

High ionic conductivity in solid electrolytes is dependent on the existence of features that interrupt the periodic nature of host materials. Here, the authors enrich the defect chemistry in a typical Li-ion conductor, reporting atom-thick planar defects that form closed loops and trap mobile species.

## Introduction

Compared to the flammable organic liquid electrolyte in current commercial Li-ion batteries, solid electrolytes can greatly alleviate the safety issues, and also allow for further increase in energy density^1–4^. However, developing highly conductive solid electrolytes with comparable ionic conductivities to liquid electrolytes has been a grand challenge^3–5^. In order to effectively achieve this goal, a large body of research has been dedicated to studying the ionic transport mechanisms of solid electrolytes^3–5^.

Fully comprehending the Li-ion transport mechanism in solid electrolytes requires in-depth studies not only to the periodic lattice, but also to structural features that deviate from the overall periodicity (referred to as “non-periodic features” below)^3,4,6,7^, such as point defects and grain boundaries. Since the existence of perfect, defect-free single crystals is forbidden by thermodynamics^8,9^, solid electrolytes, like most materials, inevitably possess many non-periodic features^7,9,10^, and their impact on ionic transport is known to be crucial^7,11–13^. For example, grain boundaries can substantially lower the total ionic conductivity (frequently by orders of magnitude) in many solid electrolytes, including Li_3_OX (X = Cl or Br) anti-perovskites^14^, Li_7_La_3_Zr_2_O_12_-based garnets^15,16^, Li_0.33_La_0.56_TiO_3_ (LLTO)-based perovskites^6^, and NASICON-type superionic conductors^4,6^. The introduction of interstitial Li^+^ as point defects may often trigger the cooperative knock-on like mechanism^17,18^ and greatly enhance the conductivity, as exemplified by Li_4_SiO_4_–Li_3_PO_4_^17^, cubic Li_7_La_3_Zr_2_O_12_^19,20^, Li_1+*x*_Al_*x*_Ti_2-*x*_(PO_4_)_3_^21^, and LiTaSiO_5_^21,22^. The H_Li_ substitutional defects caused by hydration were found to strongly affect Li-ion migration in Li_3_OCl^23^ and Al-doped Li_7_La_3_Zr_2_O_12_^24^. The lithium-halide Schottky defect pairs may greatly facilitate Li-ion transport in multiple Li-rich anti-perovskites^23,25^. Even the non-periodic features not directly involving charge carriers can significantly affect the ionic conductivity through the mixed polyanion effect^26^ and the plastic crystal behaviors like rotational motions of polyanions^27^. Clearly, before the Li-ion transport mechanism can be properly established, the non-periodic features must be thoroughly investigated first.

However, presently the understanding on the non-periodic features in Li-ion conductors is still limited. First of all, for many non-periodic features the atomistic mechanism controlling Li-ion migration is not well comprehended. A typical example is the grain boundaries. The studies that directly probed the atomic-scale mechanism of grain-boundary Li-ion transport are limited, and most of them were reported only recently^14,15,28–30^. Dawson et al. directly simulated the grain boundaries in Li_3_OCl to clarify their role in ionic transport, and successfully reconciled the discrepancies among previous experimental and computational studies^14^. Using a novel microscale strategy for simulation, they also elucidated the different contributions of grain-boundary resistance in sulfide and oxide solid electrolytes^28^. Additionally, the computational study of Yu et al. discovered that certain types of grain boundaries in Li_7_La_3_Zr_2_O_12_ may even facilitate ionic transport, and strategies for optimization was proposed accordingly^15^. These recent works provided valuable insights into both the diffusion mechanisms and conductivity improvement, but similar investigations remain scarce in literature. In order to comprehensively understand the influence of non-periodic features, more of such in-depth studies are needed. Secondly, so far only a few types of non-periodic features like point defects and grain boundaries have received attention. Nevertheless, many other non-periodic features, e.g., dislocations, stacking faults, and twin boundaries, could also be intrinsically existent in solid electrolytes^9,31^, and some of them might significantly influence the Li-ion migration too. Before the ionic transport mechanism can be fully understood, identifying these relevant non-periodic features and thoroughly understanding their influence on Li-ion migration would be indispensable. Regardless, the highly localized nature and low volume fraction of most non-periodic features^9^ make such exploration quite challenging^3,6,7^. Presently, the non-periodic features involved in most mechanistic studies are still limited to a few types like point defects and grain boundaries^3,4^.

Here, we discover an additional type of non-periodic feature that significantly influences Li-ion migration, and its role in ionic transport is unravelled at the atomic scale by combining advanced electron microscopy and ab initio calculations. In a prototype solid electrolyte Li_0.33_La_0.56_TiO_3_ (LLTO)^3,32^, closed loops formed by a kind of single-atom-layer 2D defect are found ubiquitous by electron microscopy observation; a term “single-atom-layer trap” (SALT) is coined to describe such defect loops. Based on the experimentally determined defect structure, ab initio calculations unambiguously demonstrate that the SALTs, although never discussed in previous mechanistic studies, prevent large volumes of materials from participating in ionic transport, and severely degrade the total conductivity.

## Results

### Spotting SALTs in the lattice

**Fig. 1:**
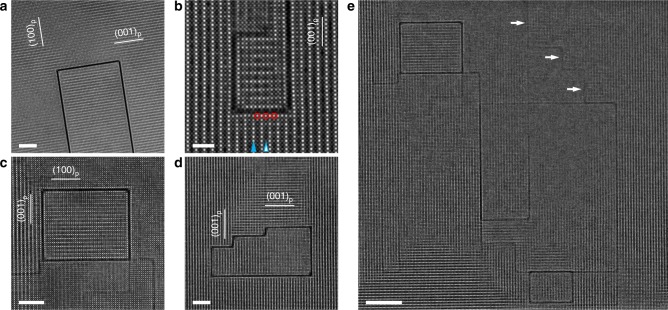
Single-atom-layer traps (SALTs) in LLTO. **a**–**d** HAADF-STEM images of the 2D defects that formed SALTs. The LLTO planes parallel with the 2D defects are marked directly in images. A La-rich A-site layer and a La-poor one in LLTO were indicated by solid and empty arrows, respectively, in **b**. The red circles indicated three A-site atomic columns that were located in the La-poor layer of LLTO but became La-rich because of the neighboring 2D defect layer. **e** Ubiquitous presence of SALTs. The bright arrows indicated three 2D defects that appeared less dark due to the coexisting LLTO in their atomic planes. The scale bars in **a**, **b**, **c**, **d** and **e** are 5 nm, 2 nm, 5 nm, 5 nm and 10 nm, respectively.

In order to investigate the non-periodic features that are relevant to ionic transport, a prototype solid electrolyte, Li_0.33_La_0.56_TiO_3_ (LLTO), was selected for study. Crystallizing in the perovskite structure, it shows a very high bulk conductivity of 10^−3^ S cm^−1^, approaching that of liquid electrolytes (10^−2^ S cm^−1^)^32,33^. The LLTO ceramics used in the present work were prepared by the common sintering method^7^. The inductively coupled plasma (ICP) spectroscopy suggested that the molar ratio of Li:La:Ti was 0.325:0.552:1, nearly identical to the nominal stoichiometry 0.33:0.56:1. The x-ray diffraction pattern (Supplementary Fig. 1) suggested that the ceramics were phase-pure with the tetragonal perovskite structure, consistent with those prepared under the same conditions^7,32^. Besides, the ionic conductivities measured by both the AC (Supplementary Figs. 2 and 3) and DC methods (3.55 × 10^−5^ S cm^−1^) also agreed very well with the reported values in literature^32–35^. However, atomic-resolution scanning transmission electron microscopy (STEM) observation spotted a large population of single-atom-layer 2D defects that had never been considered in previous mechanistic studies. Figure 1a shows a representative high-angle annular dark-field (HAADF) STEM image of such defects. Within the lattice of LLTO (regions with brighter contrast), dark straight lines were clearly visible. Given that STEM images are essentially the 2D projection of 3D objects^36^, the defects corresponding to these dark lines were actually atomic planes parallel with the observing direction, i.e., <100>_p_ of LLTO (the subscript p refers to the prototype perovskite unit cell). Since the image intensity of HAADF-STEM reflects the average atomic number^36^, the observed dark planes must exhibit a very different composition from LLTO. It should be noted that these defects were not caused by the specimen thinning procedure that is required for high-quality STEM imaging. Supplementary Fig. 4 shows the TEM images of “pristine” LLTO particles, which were obtained by manually crushing the sintered LLTO ceramic and did not undergo any thinning procedure. Despite the low image quality caused by the large specimen thickness, the defects were still spotted for multiple times, indicating that they are intrinsically existent within the material. Although these defects had never been investigated in detail before, they were surprisingly easy to find. A few more examples are displayed in Fig. 1b–d. After examining a large number of them, we discovered three important characteristics. First of all, they may only lie within one of the {001}_p_ planes of LLTO (Fig. 1a–d). As a result, the interconnected 2D defects were always perpendicular to each other. Secondly, the presence of the 2D defect appeared to influence the composition of its nearest neighboring LLTO planes. LLTO is characterized by the alternate stacking between La-rich and La-poor A-site layers, which would appear as rows of bright and dark spots in the HAADF-STEM images, respectively^7^; in Fig. 1b, a La-rich layer and a La-poor one were pointed by solid and empty arrows, respectively. Regardless, the A-sites closest to the defect always showed brightness (and thus composition) nearly identical to that of La-rich ones in bulk LLTO, even if some of them were located in the La-poor layer and should have been dark. In Fig. 1b, a few examples of such A-sites were circled in red. Last but not least, most (if not all) of the defects formed “closed loops”, isolating a volume of LLTO from the rest of the grain. Actually, besides the defects in Fig. 1c and d, those in Fig. 1a, b also formed closed loops, but they were too big to be shown completely at high magnifications. Such loops were so ubiquitous that they even shared edges with each other from time to time (Fig. 1e). Among the defects constituting these loops, some were coexisting with LLTO in the defect plane, and thus appeared less dark due to the brighter contrast of LLTO (a few examples pointed by bright arrows in Fig. 1e). In addition to the two {001}_p_ planes parallel with the viewing direction <100>_p_ in Fig. 1, the third {001}_p_ that coincided with the viewing plane could also contain 2D defects: after all, such features can be parallel with any of the three {001}_p_ planes (Fig. 1a–d). Among these three differently oriented 2D defects, any two could and would almost always form closed loops with each other (in fact, the “stand-alone” 2D defect that did not form closed loops with others was barely observed). Consequently, although only the defects in two of the three {001}_p_ planes can appear edge-on and be visible along the viewing direction <100>_p_ in Fig. 1, it is highly likely that defects also existed in the third {001}_p_ plane, i.e., the viewing plane, to form closed loops with the ones appearing edge-on in the images. That is, the 2D defects in all the three {001}_p_ planes would often (if not always) enclose 3D volumes together by forming the observed loops. It should be noted that the 2D defects here may not be considered as a special case of grain boundaries, as the loops formed by them actually existed within individual grains (Supplementary Fig. 5). Besides, as shown in Supplementary Fig. 5a, grain boundaries lacked many of the aforementioned characteristics for 2D defects, e.g., lying exclusively in {001}_p_, being visible only along certain orientations, and always forming right angle with each other. Therefore, although the observed defect may enclose 3D volumes like grain boundaries, it is a fundamentally different non-periodic feature. We coined a term called “single-atom-layer trap” (SALT) to describe the closed loops formed by these defects. Clearly, the atomic framework for Li-ion migration, as shown in Fig. 1e, cannot be fully described without taking SALTs into account.

### Composition and structure of the 2D defects in SALTs

**Fig. 2:**
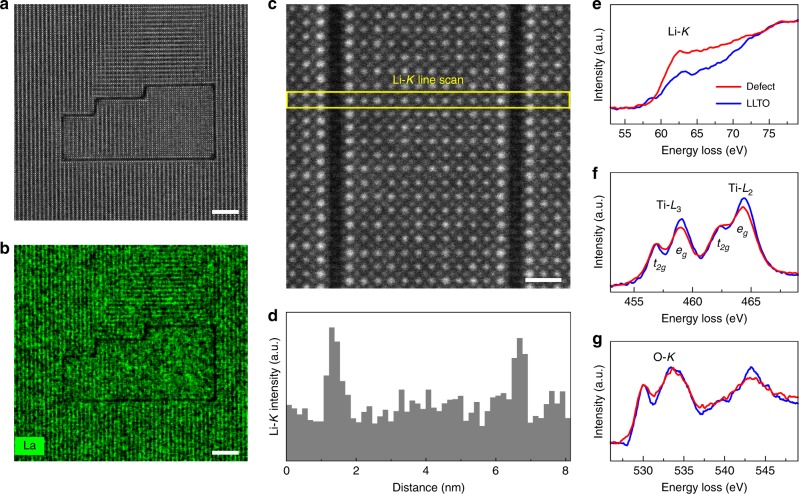
EDX and EELS analyses of the 2D defects. **a**, **b** HAADF-STEM image (**a**) and the corresponding EDX mapping result (**b**) of a SALT formed by the 2D defects. The scale bars in **a** and **b** are both 5 nm. **c**, **d** HAADF-STEM image (**c**) and the Li-*K* intensity profile (**d**) obtained via EELS line scan in the region indicated by the yellow rectangle in **c**. The scale bar in **c** is 1 nm. **e**–**g** EELS results of Li-*K* (**e**), Ti-*L*_2,3_ (**f**), and O-*K* (**g**) for the 2D defect and bulk LLTO.

Before the role of SALTs in ionic transport can be studied, the composition and structure of the 2D defects constituting them must be unraveled first. To start with, the energy-dispersive X-ray spectroscopy (EDX) was performed on a SALT shown in Fig. 2a. In the mapping result of La (Fig. 2b), a dark loop with exactly the same geometry as the SALT was immediately revealed, indicating that the La content was negligibly small. This is consistent with the HAADF-STEM imaging: considering that La is the heaviest element in LLTO and that HAADF-STEM reflects average atomic numbers^36^, the absence of La in SALTs should naturally lead to dark image contrast. Beyond La, the variation of other elements was studied by electron energy loss spectroscopy (EELS), which is more sensitive to light elements^37,38^. In the region shown in Fig. 2c, the Li-*K* signal was collected in an EELS line scan running across two adjacent 2D defects. In order to confirm that the ~62 eV signal acquired during the line scan is indeed Li-*K* rather than Ti-*M*_1_^39^, EELS was performed to the La-rich (Li-poor) and La-poor (Li-rich) layers of LLTO (Supplementary Fig. 6a), which differed greatly in Li content but exhibited the same Ti content^7,34,40^. As shown in Supplementary Fig. 6b, the 62 eV signal was found much stronger in the Li-rich layer, instead of showing similar intensities in both layers like the Ti content. Consequently, the 62 eV signal can be safely designated as Li-*K*. In the EELS line scan result (Fig. 2d), the integrated intensity of Li-*K* clearly peaked at both defects, indicating an enrichment of Li. In addition to the content, the electronic environment of Li was also quite dissimilar between LLTO and the defect, as reflected by their different Li-*K* fine structures (Fig. 2e)^37^. As for Ti and O, no significant content fluctuation was observed across the defects (Fig. 2f–g), but the EELS fine structures disclosed interesting information. The Ti-*L*_2,3_ white lines are sensitive to the oxidation state of Ti, whose variation would be reflected in the *L*_2_/*L*_3_ intensity ratio and peak positions^41,42^. Figure 2f suggested that the defects and LLTO were virtually the same in both aspects. As such, the valence of Ti should remain 4+ within the defects. Additionally, the *t*_2*g*_/*e*_*g*_ peak splitting of Ti-*L*_2,3_ in LLTO also existed in the defect. This splitting arose from the bonding of Ti with its six neighboring O^6,42^, so most likely the TiO_6_ octahedra similar to those in LLTO were present in the defects too. However, the octahedra in the latter should be distorted in a slightly different way from the former, as their *t*_2*g*_/*e*_*g*_ intensity ratios were not exactly the same^42^. Finally, the comparison of O-*K* fine structures suggested that the bonding of O with adjacent cations within defects should also be distinct from that in LLTO^37^. According to the EDX and EELS analyses presented above, the 2D defect should essentially be a single-atom-layer Li-Ti-O compound that was Li-rich and possessed TiO_6_ octahedra with Ti^4+^.

**Fig. 3:**
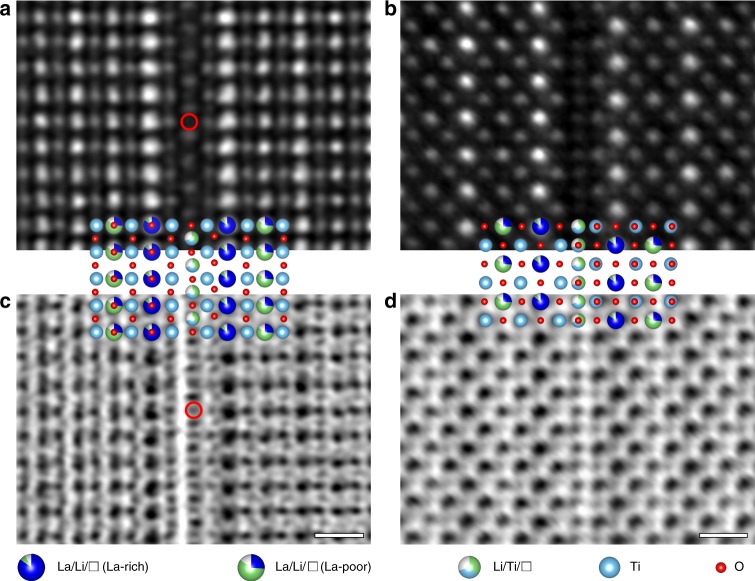
Structural analysis of the 2D defects. **a**, **c** HAADF-STEM (**a**) and ABF-STEM (**c**) images of the defect when the adjacent LLTO was oriented along $[1\bar 10]_p$. An atomic column consisting of O only was circled in red in **a** and **c**. **b**, **d** HAADF-STEM (**b**) and ABF-STEM (**d**) images of the defect when the adjacent LLTO was oriented along $[010]_p$. The scale bars in **c** and **d** are both 5 Å.

Based on these results, the structure of the defect was probed by atomic-resolution STEM observation. The study began with the configuration of LLTO unit cells at different sides of the defect. When LLTO was aligned along $[1\bar 10]_p$ (Fig. 3a), the perovskite unit cells at one side of the defect appeared to match with those at the other. That is, if the defect were replaced by an ordinary layer of LLTO, these unit cells would form a continuous, perfect lattice. Nevertheless, when LLTO was oriented along $[010]_p$ (Fig. 3b), a mismatch by half of the lattice parameter was observed. The difference between images along $[1\bar 10]_p$ and $[010]_p$ indicated that the LLTO unit cells at one side of the 2D defect must be shifted with respect to those at the other by $1/2[\bar 110]_p$, as shown in Supplementary Fig. 7a. In this way, when multiple unit cells were present, projecting the entire 3D object to the viewing planes in Fig. 3a, b, as shown in Supplementary Fig. 7b, c, respectively, would precisely produce the appearances described above. With the configuration of neighboring LLTO unit cells determined, the structure of the 2D defect itself may be analyzed. It was found that, no matter whether the observation was performed along $[1\bar 10]_p$ (Fig. 3a, c) or $[010]_p$ (Fig. 3b, d) of LLTO, the defect always exhibited well defined, highly ordered atomic columns like those in a periodic lattice. Therefore, albeit single-atom-layer thick, it may very likely be isostructural with a known compound that was capable of forming epitaxy with perovskites. Combining this clue with the characteristics learnt from Fig. 2, i.e., Li-Ti-O compound, TiO_6_ octahedra, Ti^4+^, etc., the rock-salt-structured Li_2_TiO_3_ with $Fm\bar 3m$ space group^43^, i.e., γ-Li_2_TiO_3_, immediately arose as a highly probable candidate. It is well known that the rock-salt structure can easily interface with perovskites in an epitaxial manner^44,45^. Besides, the rock-salt γ-Li_2_TiO_3_ did exhibit TiO_6_ octahedra, and the Ti valence was also 4+. If it interfaced epitaxially with LLTO, the <110> and <010> would be parallel with <110>_p_ and <010>_p_ of LLTO, respectively, according to the reported epitaxial relationship^44^. Therefore, should the 2D defect observed here be a single-atom-layer γ-Li_2_TiO_3_ and follow such orientations, it may only be the atomic plane containing both <110> and <010>, i.e., the {001} plane. In order to verify whether the defect really took this structure, we constructed the atomic model accordingly and compared it with the STEM images in Fig. 3. The comparison was first made along the $[1\bar 10]_p$ zone axis of LLTO. When the LLTO unit cells in the atomic model were aligned with the HAADF-STEM image (Fig. 3a), the Li/Ti columns of the defect matched very well with the bright spots. Since HAADF-STEM cannot easily detect light elements, the locations corresponding to pure O columns (one of them circled in red) in the atomic model barely showed any contrast. However, when examined by ABF-STEM (Fig. 3c), which is sensitive to elements with small atomic numbers^46^, the O columns were clearly visualized at expected positions too (one of which circled in red). In order to further confirm the speculated defect structure, we also made the comparison when LLTO was aligned along $[010]_p$. To this end, the entire atomic model, i.e., both the defect and the neighboring LLTO unit cells, in Fig. 3a, c was rotated around $[001]_p$ of LLTO by 135°, so that LLTO was tilted from $[1\bar 10]_p$ to $[010]_p$. Then, this re-oriented atomic model was compared with the HAADF- and ABF-STEM images taken along $[010]_p$ of LLTO (Fig. 3b, d, respectively). Again, they matched perfectly with each other. Therefore, it should be safe to conclude that the speculation above was correct: the 2D defect was indeed isostructural with the {001} plane of the rock-salt-structured γ-Li_2_TiO_3_.

Nevertheless, exhibiting the rock-salt structure does not necessarily mean that the stoichiometry is precisely Li_2_TiO_3_. In fact, the composition of the 2D defect must ensure an overall charge balance. This was schematically illustrated in Supplementary Fig. 8. First of all, the unit cell of LLTO consists of one La-rich, one La-poor, and two Ti-O planes parallel with (001)_p_. Each Ti-O plane shows a Ti/O ratio of 1/2, and is thus charge balanced. The compositions of La-rich and La-poor layers here cannot be determined precisely, but an acceptably good approximation can be found in a recent neutron diffraction study^40^, where the stoichiometry of the material under study (confirmed by ICP analysis) was also Li_0.33_La_0.56_TiO_3_. In this way, the La-rich and La-poor layers within the unit cell shown in Supplementary Fig. 8 can be estimated to carry +0.62 and −0.62 charges, respectively, which set off each other to maintain the charge balance of the unit cell. Since the A-site layers neighboring the defect are nearly identical with the La-rich ones in bulk LLTO, as reflected by their similar intensities in the Z-contrast images (Fig. 1), the in-between 2D defect must exhibit the −0.62 charge that should have been carried by a La-poor layer. Such a negative charge entailed that the 2D defects, unlike the charge balanced γ-Li_2_TiO_3_, should contain cation vacancies. Assuming these vacancies arising from Li deficiency only, each Li/Ti/vacancy site in Fig. 3 and Supplementary Fig. 8 should contain 0.37 Li, 0.33 Ti, and 0.30 vacancies to reach −0.62 charge within the “unit cell” of the defect plane (such a “unit cell”, as shown in Supplementary Fig. 8, consisted of 2 Li/Ti/vacancy and 2 O sites). In this configuration, the Li concentration within the defect layer may thus be calculated to be around 2.23 times of that in bulk LLTO. Interestingly, this was quite close to the value obtained by the quantification analysis of Li-*K* edges in Fig. 2e (2.06), indicating the stoichiometry raised above, albeit a rough estimation, should not deviate much from reality. Additionally, we also simulated the STEM images using this atomic model, and the results (Supplementary Fig. 9) agreed well with the experimental observation in Fig. 3 too (the minor discrepancies arose from the distractions that are inevitable in experimental imaging but cannot be precisely incorporated in simulation, e.g., drifting, inaccuracy of specimen alignment, and uncertainty of the specimen thickness). Therefore, the 2D defect should be a single-atom-layer compound that is isostructural with {001} of the rock-salt γ-Li_2_TiO_3_ and exhibits an estimated composition of [Li_0.37_Ti_0.33_O]^0.31−^ (or [Li_1.11_TiO_3_]^0.93−^). The atomic configuration within the defect plane was shown schematically in Supplementary Fig. 10.

**Fig. 4:**
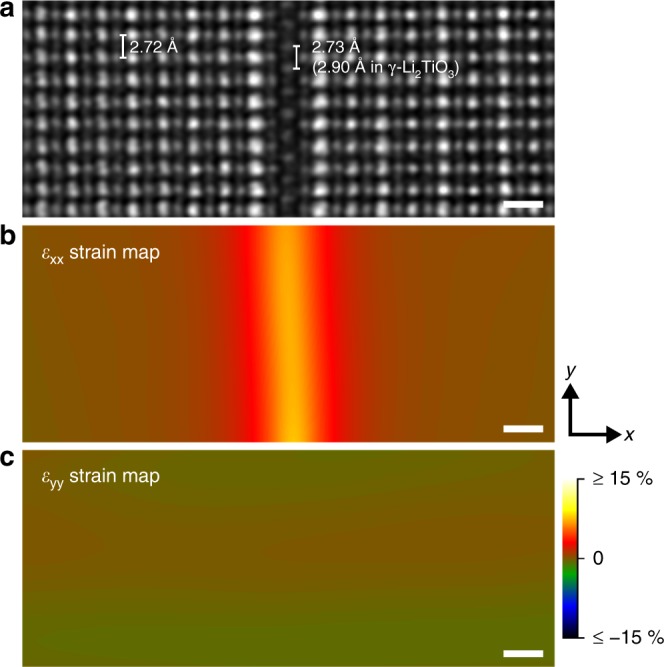
Strain variation across the 2D defects. **a** HAADF-STEM image of a 2D defect with the adjacent LLTO oriented along $[1\bar 10]_p$. **b**, **c** *ε*_xx_ (**b**) and *ε*_yy_ (**c**) strain maps obtained by performing GPA on the image in **a**. The scale bars in **a**–**c** are all 5 Å.

In order to investigate how the 2D defect was connected with LLTO, geometric phase analysis (GPA)^47^ was performed. Under most circumstances, the epitaxial growth of one phase on the other would be accompanied by periodic misfit dislocations at the interfaces, so that their lattice mismatch can be reconciled^48^. Surprisingly, this phenomenon was not observed at the 2D defect, even if the lattice parameter of bulk γ-Li_2_TiO_3_ (4.10 Å) is much larger than that of LLTO (3.87 Å). According to the GPA results (Fig. 4), only *ε*_*xx*_ maximized at the 2D defect, reflecting a local enlargement of the distance between atomic planes perpendicular to the defined *x* direction, while *ε*_*yy*_ did not show any fluctuation that can indicate the existence of misfit dislocations. The interatomic distances were investigated to unravel the mechanism behind. It was found that the distance between two neighboring atomic columns of the 2D defect in Fig. 4a was only 2.73 Å. This value was much smaller than the same spacing in bulk γ-Li_2_TiO_3_ (2.90 Å) but matched quite well with that in LLTO (2.72 Å). The compactness of single-atom-layer [Li_1.11_TiO_3_]^0.93−^ with respect to γ-Li_2_TiO_3_ may be attributed to its Li deficiency, or compression from the surrounding LLTO, or both. Regardless, thanks to such a contraction of interatomic distances, the 2D defects were able to fit smoothly into the LLTO lattice, without involving any dislocations.

### SALTs versus Li-ion transport

**Fig. 5:**
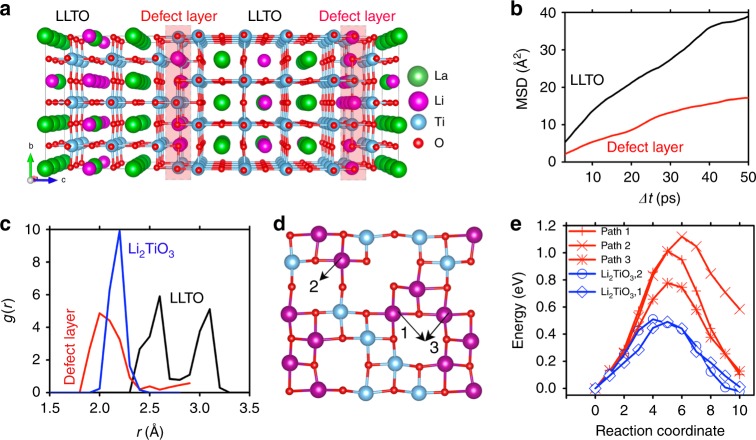
First principles atomistic modeling of the 2D defects. **a** Atomistic model used for calculations. **b** MSD of Li ions in bulk LLTO versus that within (and also parallel with) the 2D defect layer from AIMD simulations at 2000 K. **c** Radial distribution functions *g(r)* of Li-O bonds in the 2D defect layer, β-Li_2_TiO_3_, and bulk LLTO. The 2D defect layer has shorter Li-O bond length than bulk LLTO and β-Li_2_TiO_3_. **d** Li-ion migration pathways selected for NEB calculations in the 2D defect layer. **e** Migration energy profiles of selected Li-ion pathways in the 2D defect layer (red data, pathways indicated in **d**) and of similar pathways within the (202) plane of β-Li_2_TiO_3_ (blue data, pathways indicated in Supplementary Fig. 12).

In order to investigate the influence of SALTs on ionic transport, the interplay between individual 2D defects and Li-ion migration must be understood first. To this end, we constructed the atomic model for first-principles calculations based on the defect structure determined above. Since the LLTO lattice at one side of the defect was shifted by $1/2[\bar 110]_p$ with respect to that at the other (Supplementary Fig. 7a), the individual LLTO/defect/LLTO sandwich does not satisfy the periodic boundary condition along the [001]_p_ direction of LLTO, and thus cannot be used directly for calculations. As a result, we constructed the atomic model via the back-to-back combination of two LLTO/defect/LLTO sandwiches instead. In such a model (Fig. 5a), the LLTO lattice on the right side was shifted by $1/2[\bar 110]_p$ with respect to that in the middle, which was also shifted by $1/2[\bar 110]_p$ with respect to that on the left side. Consequently, the LLTO lattice on the right side was shifted by $[\bar 110]_p$ with respect to that on the left, satisfying the periodic boundary condition. With the atomic model constructed this way, the experimentally determined defect structure was confirmed by density functional theory (DFT) calculations.

Ab initio molecular dynamics (AIMD) simulations were then performed to investigate the Li-ion transport behavior. During the simulation, we observed no Li-ion diffusion crossing between the 2D defect layer and the neighboring LLTO. This can be explained by the La-rich LLTO layers adjacent to the 2D defect, which are known to be Li-ion-blocking due to the strong repulsion of La^3+^ and limited number of vacancies for Li-ion hopping^49,50^. Because of these non-conductive atomic layers, Li-ion diffusion cannot happen in the perpendicular direction across the defect layer. In the directions parallel with the defect layer, the Li-ion diffusion within the 2D defect was found much slower than that in the bulk phase of LLTO, as measured by the Li-ion mean square displacement (MSD) over time (Fig. 5b). The poorer ionic transport may be attributed to the compressed volume for Li-ion migration, which resulted from the close packing of Li-O within the 2D defect. According to the calculated radial distribution functions *g*(*r*) of Li-O, the distance between Li and O was much shorter in the 2D defect than in LLTO (Fig. 5c). The resulting smaller volume increased the migration barrier for Li ions, and thus led to the poor mobility reflected in Fig. 5b. We further compared the 2D defect with β-Li_2_TiO_3_ (space group *C2/c*)^51^. This compound shows similar atomic configurations with the 2D defect in its (202) plane, and the ionic conductivity is known^51,52^; based on the Arrhenius plot between 300 and 600 °C^52^, its room-temperature conductivity can be estimated as 2.43 × 10^−12^ S cm^−1^. Compared to such a poor ionic conductor, the 2D defect, with shorter Li-O distances (Fig. 5c), shows even slower Li-ion diffusion, as confirmed by the nudged elastic band (NEB) calculations. Among the Li-ion pathways observed in AIMD simulations, three of them were selected from the 2D defect layer (Fig. 5d) to calculate the Li-ion migration energy profiles. The Li-ion migration barrier was found barely affected by the local A-site configurations in the adjacent LLTO layers (Supplementary Note 1 and Supplementary Fig. 11), but dependent on the configuration of Ti, Li, and vacancies within the defect layer. The ground-state configuration of the defect layer from our calculations exhibits a chain-like Ti configuration (Fig. 5d), which is similar to that in the (202) plane of β-Li_2_TiO_3_ (Supplementary Fig. 12). Li-ion migration across this Ti chain exhibits a high barrier of ~1.2 eV (path 2 in Fig. 5d, e), and the migration besides the Ti chain shows a barrier of ~0.8 eV (paths 1 and 3 in Fig. 5d, e). The Li-ion diffusion in the 2D defect layer was compared with that in β-Li_2_TiO_3_, where similar pathways as those indicated in Fig. 5d (Supplementary Fig. 12) were selected for study. It was found that the 2D defect layer exhibited much higher energy barriers (0.8 − 1.2 eV) than β-Li_2_TiO_3_ (0.49 − 0.53 eV, consistent with the experimental and theoretical studies in literature^53,54^). As a result, Li-ion migration within the 2D defect is likely to be slower than that in β-Li_2_TiO_3_, a material with only 2.43 × 10^−12^ S cm^−1^ conductivity^52^ (Note: this by no means suggests that the “SALT conductivity” acquired by fitting the Nyquist plot of LLTO should supposedly be lower than 2.43 × 10^−12^ S cm^−1^. The conductivity obtained from the Nyquist plot of LLTO is a fundamentally different physical quantity from what is being estimated here. The details are explained in Supplementary Note 2).

Although such an extremely slow Li-ion migration appeared to happen only in the single-atom-thick defect layers, the SALTs formed by these defects have very profound influences on the overall ionic transport. It should be noted that the LLTO atomic planes adjacent to the 2D defect always became the La-rich A-site layers (Fig. 1a–d), which were known to be Li-ion-blocking^7^. Therefore, Li-ion diffusion across the 2D defect would be even more difficult than that within the defect layer. Since the latter was already slower than 2.43 × 10^−12^ S cm^−1^, ionic transport through the defect layer should be forbidden; in fact, such a transport behavior, according to our AIMD simulation results described above, was indeed absent even at 2000 K. When these Li-ion-blocking 2D defects form SALTs to enclose 3D volumes, Li^+^ would not be able to enter or escape the regions inside. The enclosed volumes are thus isolated from the rest of the material, and can hardly participate in the overall ionic transport. Presently it is difficult to tell whether the SALTs or the grain boundaries^3,6^ are more severely impeding the ionic transport, because the reliable simulation of grain boundaries is challenging as reflected by the rareness of such studies^14,15,28,29^. Nevertheless, the Li-ion-blocking SALTs are ubiquitously present like grain boundaries, and the volumes they isolated are also considerable (the grain size is 2−4 μm, while the volume enclosed by the individual SALT varies between 15 nm and 1 μm; besides, many grains possess multiple SALTs, as shown in Supplementary Figs. 4 and 5). As a result, these features should cause massive degradation to the total ionic conductivity too. In order to evaluate the significance of such effect, we estimated the average volume fraction that was isolated by SALTs in each grain. The estimation covers all the grains that had been selected (randomly) for observation in this study, including those without SALTs; for SALT-free grains, the isolated volume fraction was counted as 0%. In this way, it was estimated that averagely 15.7 vol% of LLTO is isolated by SALTs. As demonstrated above, these isolated volumes cannot participate in the overall ionic transport, so in practice they are non-conductive, like pores in the ceramic. Consequently, although the LLTO pellet used in the present study (relative density 96.3%) only carries 3.7 vol% of pores, the presence of SALTs is equivalent to making an additional 15.1 vol% (=96.3% × 15.7%) of the ceramic into non-conductive pores. For oxide solid electrolytes, when the porosity increases from 3.7% to 18.8% (=3.7% + 15.1%), the conductivity would decrease by 1−2 orders of magnitude, regardless of the material system^55–59^. Supposedly, SALTs are causing similar conductivity degradation, so they should no longer be left unattended. The potential approaches to eliminate SALTs are yet to be explored. However, our estimated formation energies (Supplementary Note 3) suggest that the emergence of SALTs might be favored by the loss of Li and/or O during synthesis, which frequently happens due to the high sintering temperature of LLTO^34^. If future studies can develop corresponding strategies to minimize the population of SALTs, further improvement in total ionic conductivity can be expected. Clearly, non-periodic features beyond point defects and grain boundaries could be vital in both comprehending the ionic transport mechanism and optimizing the materials performances. Therefore, they must be thoroughly investigated in all of the important solid electrolyte systems.

## Discussion

In summary, we discovered an additional type of non-periodic feature that could greatly influence ionic transport, and a term “single-atom-layer trap” (SALT) was coined to describe this phenomenon. SALTs are closed loops formed by single-atom-layer 2D defects. Although this feature has never been discussed in previous mechanistic studies, our atomic-resolution STEM observation spotted numerous SALTs in a prototype solid electrolyte Li_0.33_La_0.56_TiO_3_. Based on the experimentally determined defect structure, AIMD simulations suggested that Li ions are impossible to migrate across the 2D defects constituting SALTs. Consequently, the volumes enclosed by SALTs cannot participate in the overall ionic transport. Although further study is needed to compare the effect of SALTs and that of grain boundaries^14,15,28,29^, the large population of SALTs entails that these Li-ion-blocking features must be causing considerable degradation to the total conductivity. This discovery demonstrated that non-periodic features apart from the point defects and grain boundaries could severely impact the Li-ion migration too. In order to fully comprehend the ionic transport mechanisms, similar study should be performed to other solid electrolyte systems.

## Methods

### Materials and macroscopic characterizations

The LLTO ceramics were prepared through a sol-gel approach. Stoichiometric amount of LiNO_3_ and La(NO_3_)_3_·6H_2_O were dissolved in ethylene glycol monomenthyl ether, and then mixed with tetrabutyl titanate and acetylacetone. After drying at 70 °C, the gel was calcined at 900 °C for 6 h. The calcined powder was then ball milled for 12 h, pressed into a pellet, sintered at 1350 °C for 6 h, and finally annealed at 800 °C for three days to form the dense LLTO ceramic with the tetragonal structure. To compensate for Li loss at high temperatures, the pellets were buried in powders with the same composition during the heat treatment described above. The density of the sintered pellet was determined using Archimedes’ principle. The crystal structure, stoichiometry, and ionic conductivity of the pellets were confirmed by X-ray diffraction, inductively coupled plasma spectroscopy, and electrochemical impedance spectroscopy, respectively. The ionic conductivity was also measured by the DC method using the experimental configuration reported by Inaguma et al.^35^.

### Electron microscopy

TEM specimens were prepared by mechanically thinning the LLTO ceramic followed by Ar-ion milling with liquid nitrogen cooling at around −100 °C. The ion milling was performed at 3 kV and 4 mA at first. Upon perforation, the specimen was milled at 1.5 kV and 3 mA to remove the surface amorphous layer. The ion-milled specimens were stored in 10^−5^ torr vacuum until electron microscopy observation. The STEM/EELS study was conducted on an aberration-corrected FEI Titan Themis TEM/STEM equipped with a Gatan Image Filter Quantum-965. To avoid possible electron beam damage, the microscope was operated at 200 kV with dose rates below 12 e^−^ Å^−2^ s^−1^. The STEM images presented here were Fourier-filtered to minimize the contrast noise, and such processing did not introduce any artifact that may alter our conclusions. The simulation of STEM images was performed using the QSTEM simulation package. The strain maps were calculated from the STEM image using the commercial GPA software of HREM Research^47^. The volume fraction isolated by SALTs in each grain was determined using the software ImageJ. The EELS data were acquired in STEM mode with a 5 mm aperture and an energy dispersion of 0.1 eV per channel.

### First principles computation

All of the DFT calculations were performed using the projector augmented-wave (PAW)^60^ approach using Perdew–Burke–Ernzerhof (PBE)^61^ generalized-gradient approximation (GGA) implemented in the Vienna ab initio simulation package (VASP)^62^. All structure optimization calculations were performed using the convergence parameters set of the Materials Project^63^. The defect layer was modelled in a supercell consisting of two slabs of LLTO and two defect layers using the periodic boundary condition (CIF file attached as Supplementary Data 1). From the disordered structure of LLTO and the defect layer from experiments, the atomistic configuration of Li, La, and Ti in the supercell models was generated in a two-step process by comparing and ranking distinctive configurations using the scheme as in our previous work^64^ according to electrostatic energies and DFT energies: (1) the atomistic configuration with the lowest GGA energy was selected from the DFT calculations on 100 distinctive configurations, which were obtained according to the minimum electrostatic energy out of 10000 symmetrically distinctive configurations using pymatgen^65^; (2) the same process was repeated for ordering the atomistic configuration in the 2D defect layers to obtain a low-energy structure model of the defect for further calculations. The NEB calculations were performed with the supercell model of 11.83Å x 11.75Å x 31.84Å containing 378 atoms (detailed structure described in the CIF file attached as Supplementary Data 1). The paths of the Li-ion migration in the 2D defect were chosen from those observed in the AIMD simulation. For β-Li_2_TiO_3_, the possible migration pathways similar to those in the defect layer were considered. NEB calculations were performed using nine images linearly interpolated from fully relaxed initial and final structure, and were converged within 0.01 eV Å^−1^. To accomplish longer simulation time with reasonable computational cost, we conducted AIMD simulations on a smaller supercell model of 11.83Å x 11.75Å x 16.62Å containing 202 atoms, in which the La-poor layer in the LLTO slab was removed. The AIMD simulation, which was non-spin polarized using single Γ-centered *k*-point, was performed at 2000 K with a time-step of 2 fs in NVT using a Nosé-Hoover thermostat^66^ for a total time of 80 ps. Since Li ions in the 2D defect were observed to migrate always within itself in our simulations, the Li-ion diffusivity of the 2D defect was evaluated from the displacements of all Li ions in this particular atomic layer using the scheme established in the previous study^67^.

## Supplementary information

Supplementary Information

Description of Additional Supplementary Files

Supplementary Data 1

## Data Availability

The authors declare that all relevant data are included in the paper and its Supplementary Information file. Additional data are available from the corresponding authors upon reasonable request.
