# Supplementary material for: Single-atom-layer traps in a solid electrolyte for lithium batteries
Source: Nat Commun. 2020 Apr 14;11:1828. doi: 10.1038/s41467-020-15544-x (PMC7156726; doi:10.1038/s41467-020-15544-x)
Supplement: Supplementary file 1 — Supplementary Information [file 41467_2020_15544_MOESM1_ESM.pdf]

Supplementary Information for

Single-atom-layer traps in a solid electrolyte for lithium batteries

Zhu et al.

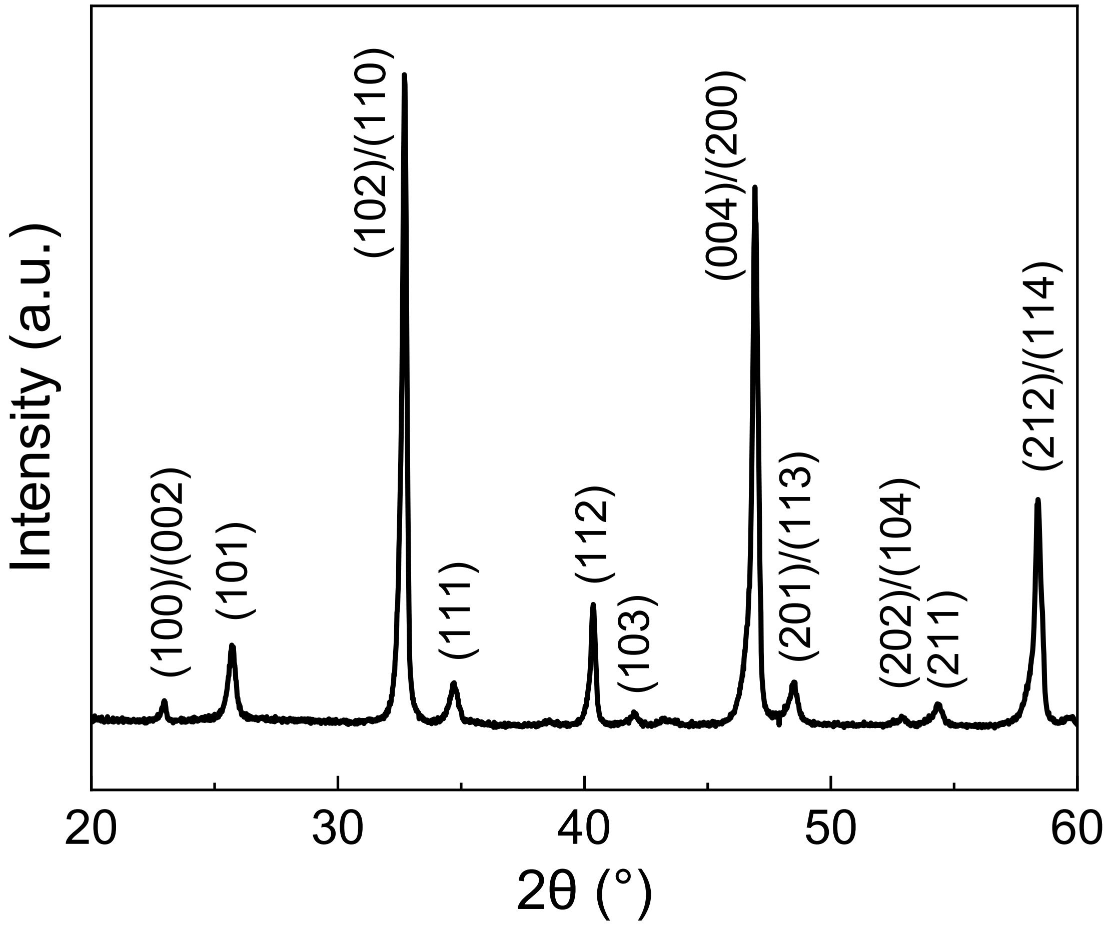

Supplementary Fig. 1 | X-ray diffraction pattern of the LLTO ceramic. The diffraction pattern can be well indexed by tetragonal LLTO (PDF 87-0935).

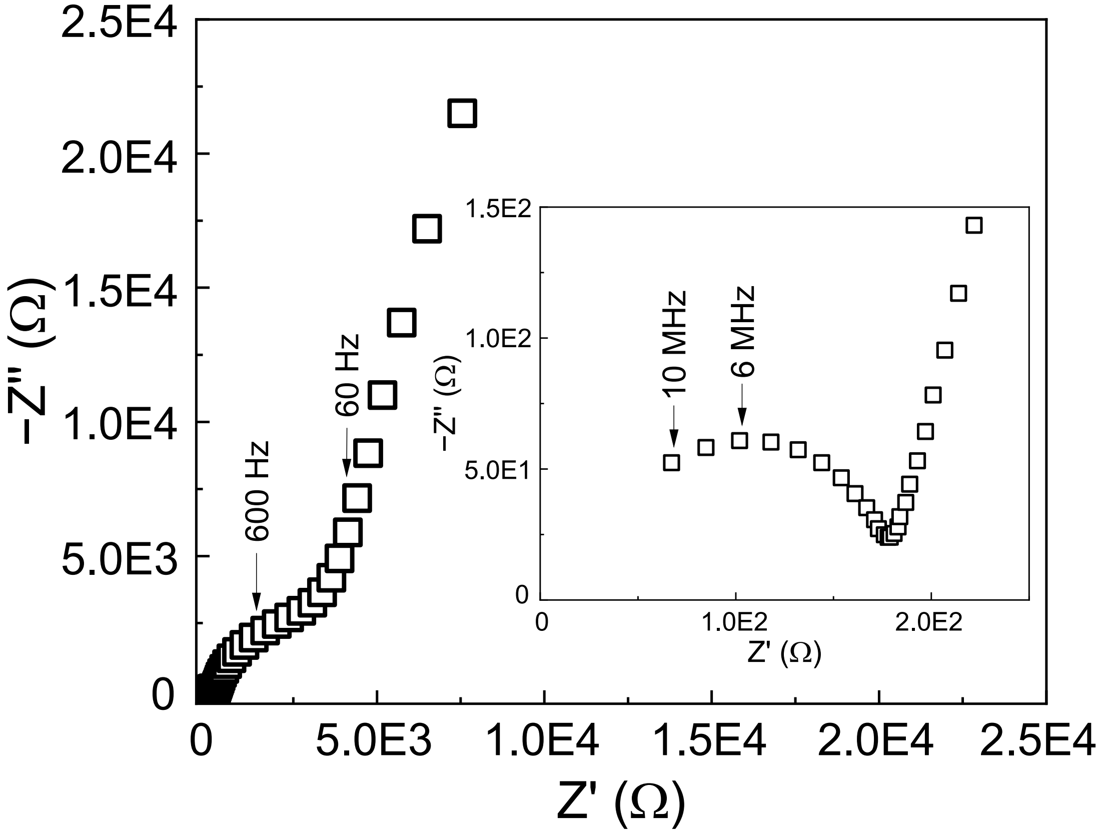

Supplementary Fig. 2 | Room-temperature Nyquist plot of the LLTO ceramic. Consistent with previous reports[1], a low-frequency semicircle at around 600 Hz and a much smaller high-frequency one at around 6 MHz (visible only in the inset) were observed. The low-frequency and high-frequency semicircles were believed to arise from the grain boundaries and the bulk, respectively, in literature[1]. The inset shows the high-frequency part of the data.

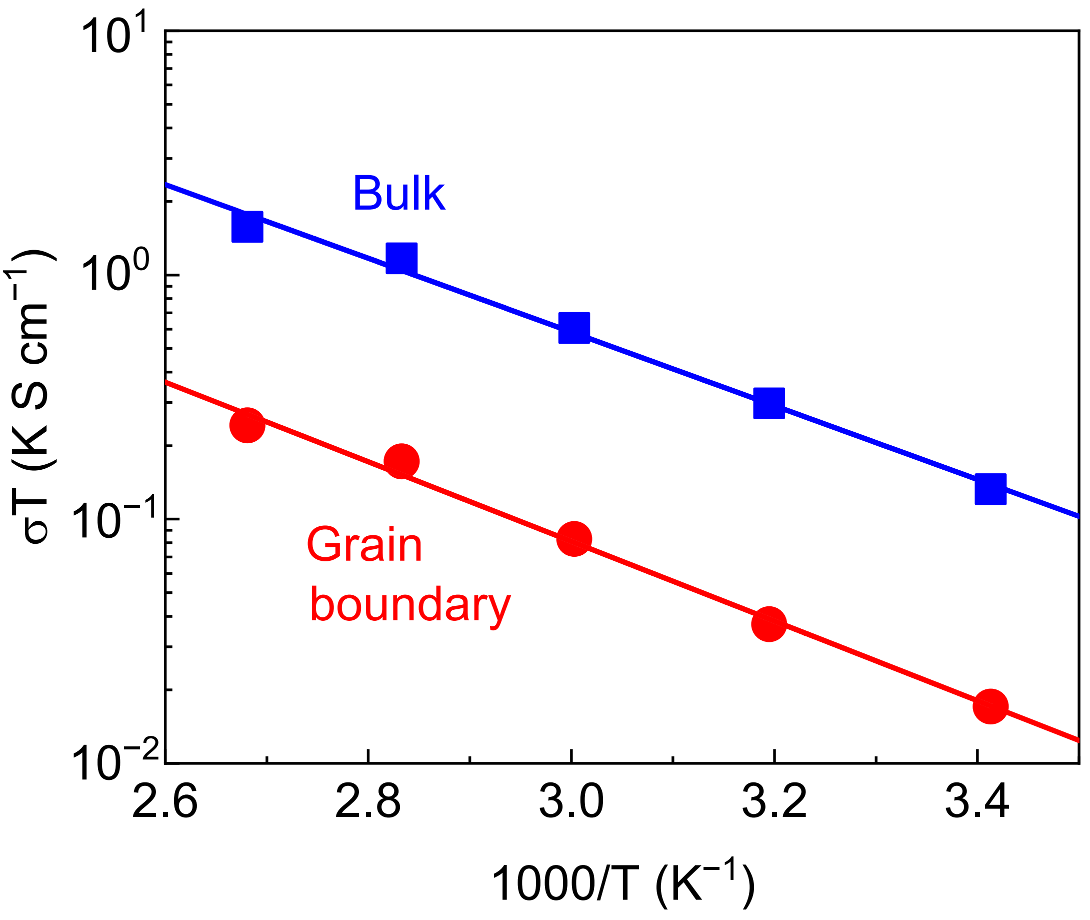

Supplementary Fig. 3 | Arrhenius plot of the LLTO ceramic. Both the bulk and grain-boundary conductivities agreed well with those in literature.

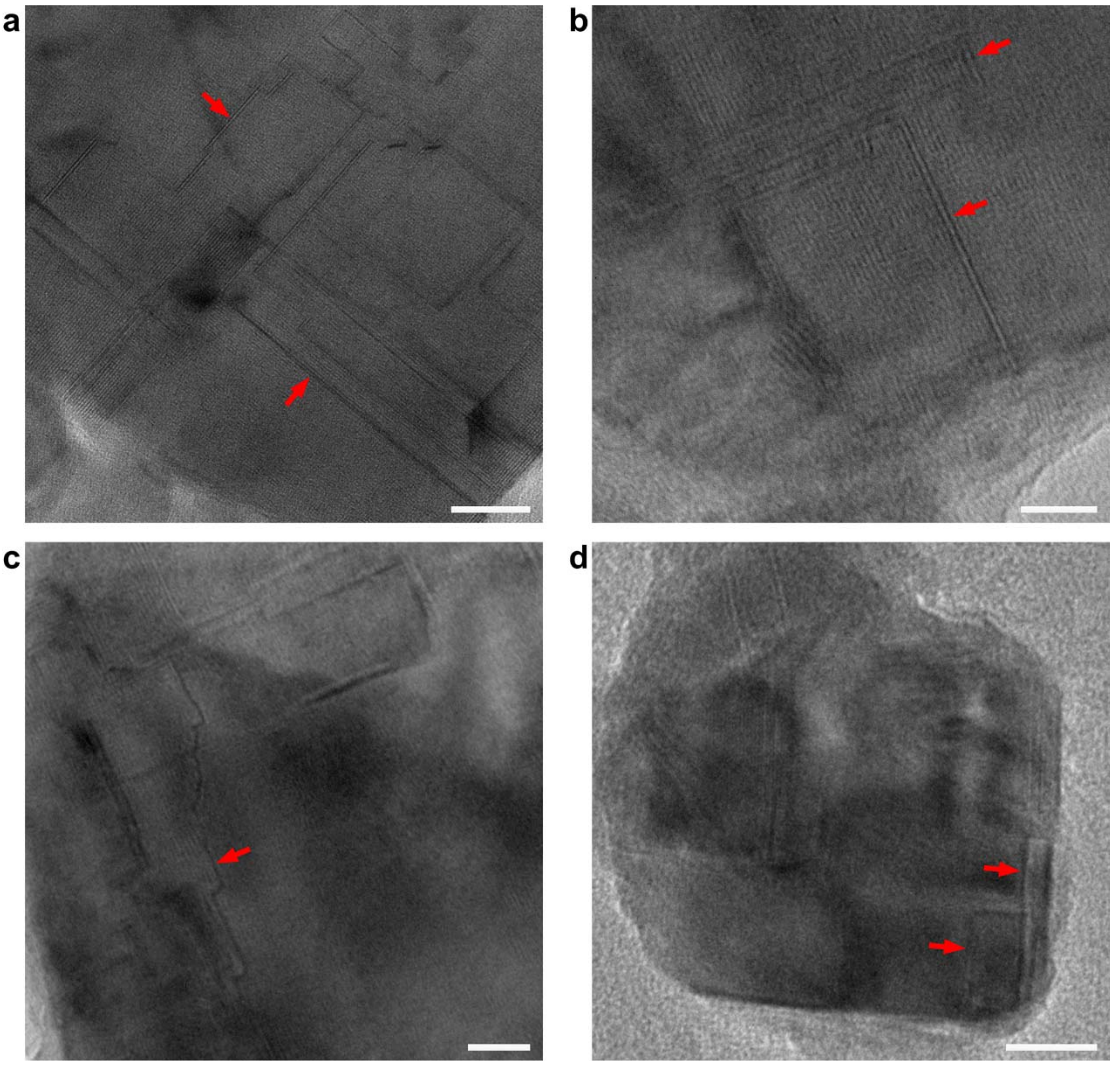

Supplementary Fig. 4 | 2D defects are not caused by the TEM specimen thinning procedures. a–d, Conventional TEM images of the “pristine” LLTO particles that were obtained by crushing the sintered ceramic. Without the TEM specimen thinning procedure, these LLTO particles still exhibited a large number of 2D defects (a few of them were arrowed in red). The scale bars in **a**, **b**, **c**, and **d** are 20 nm, 10 nm, 10nm, and 20 nm, respectively.

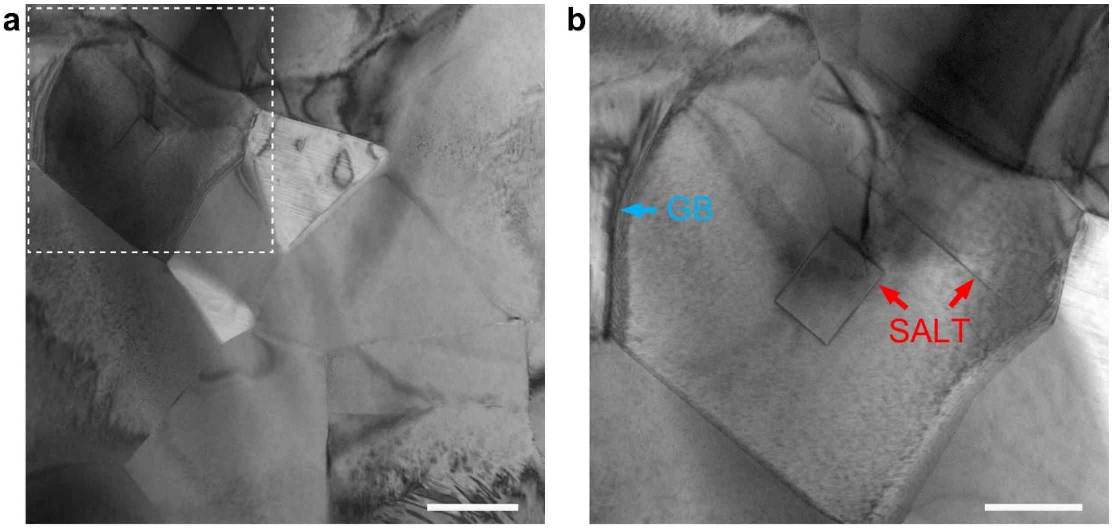

Supplementary Fig. 5 | Grain boundaries vs. the observed 2D defects. a, Low-magnification bright-field TEM image of LLTO. Among all the grains shown in this image, only one (highlighted with a dashed square) was properly oriented to make the 2D defects visible; other grains may possess 2D defects as well, but the inappropriate orientations made it impossible to visualize them. In comparison, most (if not all) of the grain boundaries are visible, regardless of the orientation. **b**, Enlarged TEM image of the region delineated with dashed lines in **a**. “GB” and “SALT” stand for “grain boundary” and “single-atom-layer trap”, respectively. The scale bars in **a** and **b** are 1 μm and 500 nm, respectively.

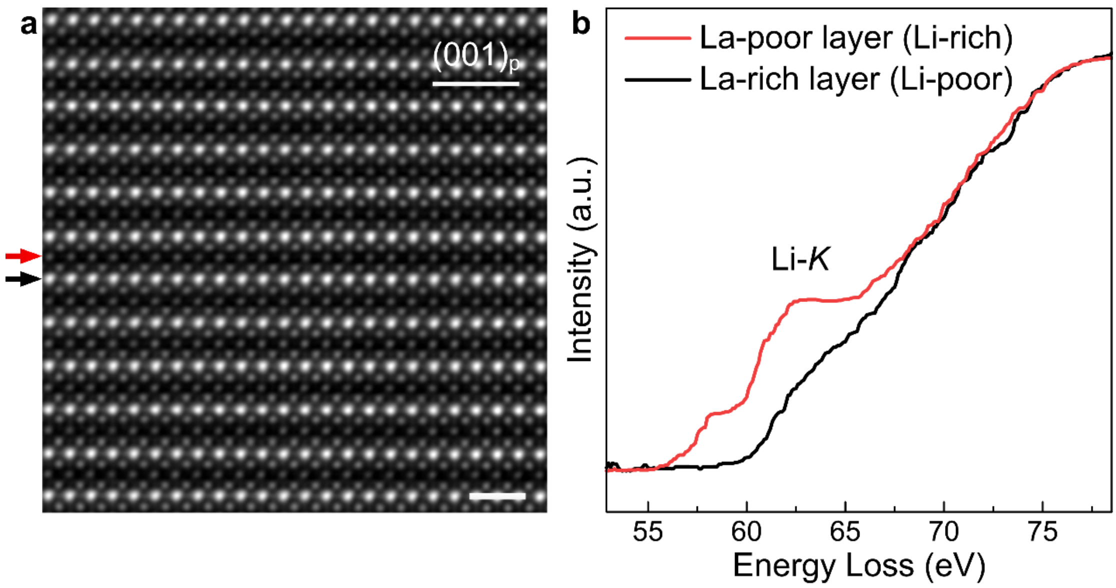

Supplementary Fig. 6 | Li-*K* signal in EELS. a, HAADF-STEM image of LLTO along $[010]_p$. A La-poor (Li-rich) atomic layer and a La-rich (Li-poor) one were arrowed in red and black, respectively. The scale bar is 1 nm. **b**, EELS results of the La-poor (Li-rich) and La-rich (Li-poor) layers in LLTO.

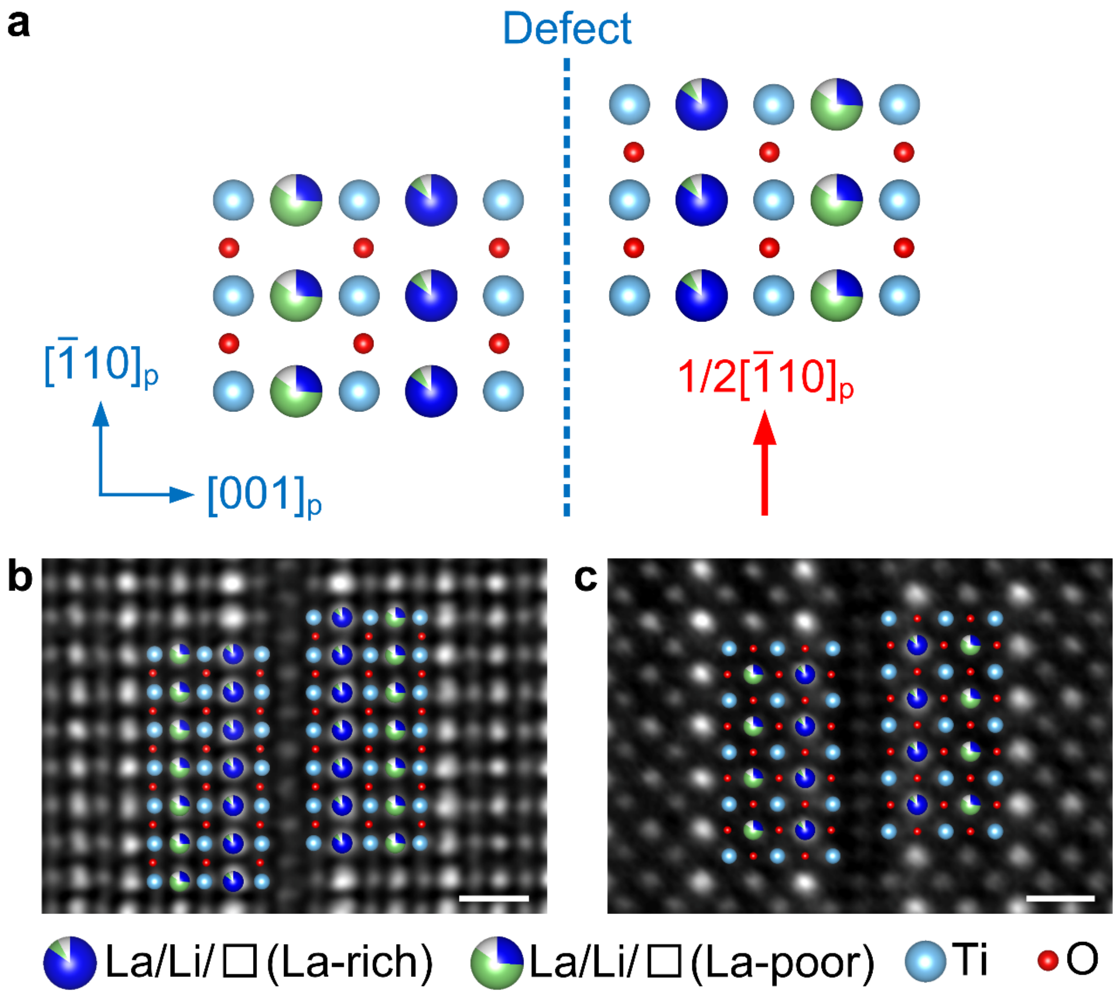

Supplementary Fig. 7 | LLTO on different sides of the defect. a, Schematic showing that the LLTO lattices on different sides of the defect are shifted by $1/2[\bar{1}10]_p$ with respect to each other. **b**,**c**, No matter whether the atomic model in **a** was oriented along $[1\bar{1}0]_p$ (**b**) or $[010]_p$ (**c**) of LLTO, it always agreed well with the corresponding HAADF-STEM images. The scale bars in **b** and **c** are both 5 Å.

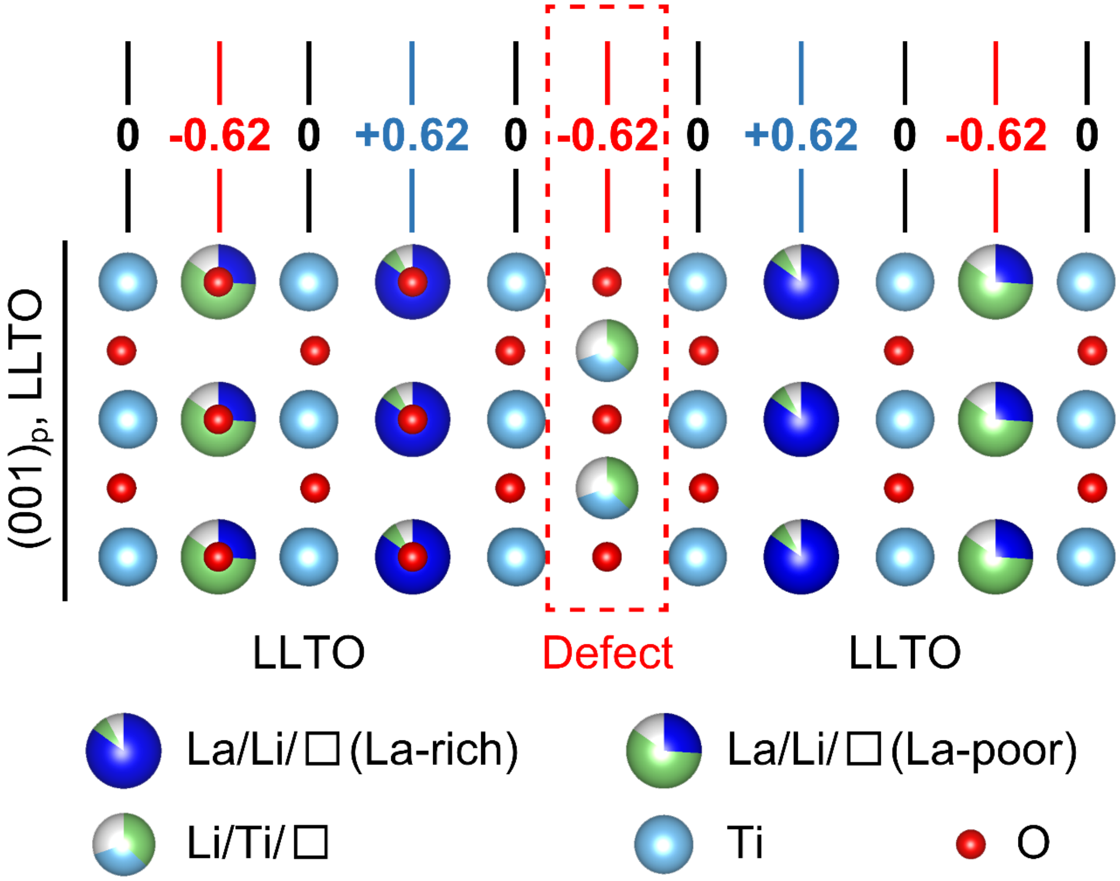

Supplementary Fig. 8 | Charge balance requirement for the 2D defect. In order to maintain the overall charge balance, each "unit cell" of the 2D defect must carry -0.62 charge.

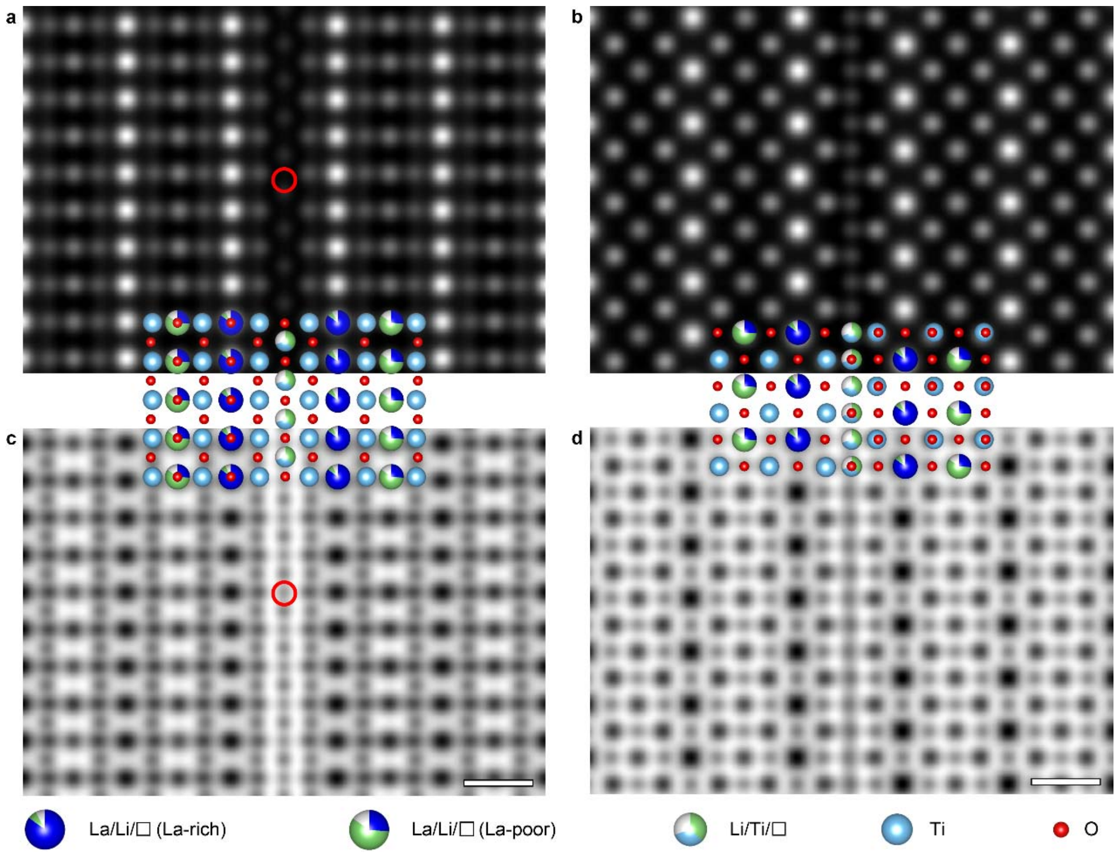

Supplementary Fig. 9 | Simulated STEM images of the 2D defect. a,**c**, Simulated HAADF- (**a**) and ABF-STEM (**c**) images of the defect when the adjacent LLTO was oriented along $[1\bar{1}0]_p$. An atomic column consisting of O only was circled in red in **a** and **c**. **b**,**d**, Simulated HAADF- (**b**) and ABF-STEM (**d**) images of the defect when the adjacent LLTO was oriented along $[010]_p$. The scale bars in **c** and **d** are both 5 Å.

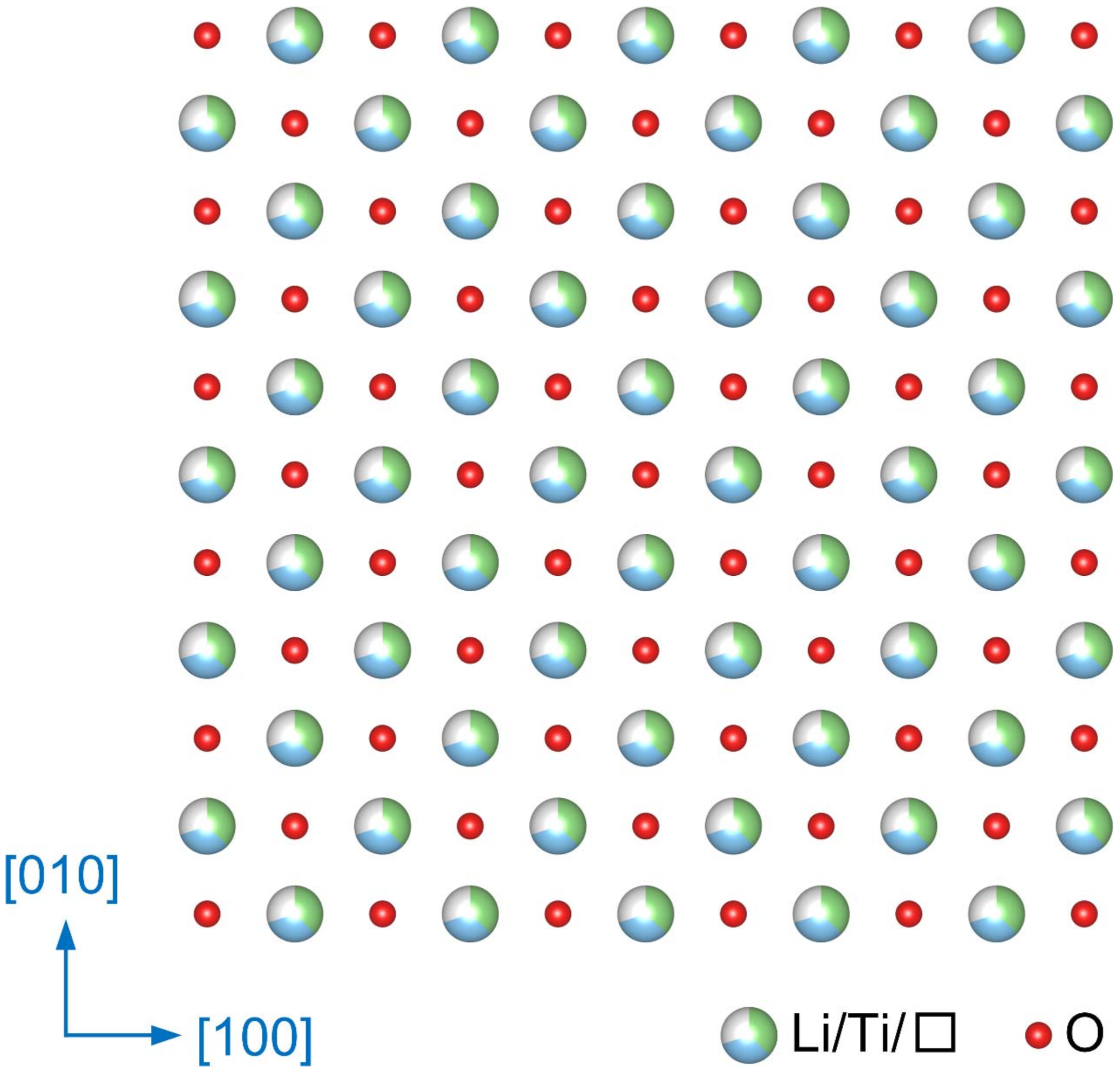

Supplementary Fig. 10 | In-plane structure of the 2D defect. The [100] and [010] axes here refer to those in the rock-salt γ-Li_2TiO_3 structure. Each Li/Ti/□ site was estimated to contain 0.37 Li, 0.33 Ti, and 0.30 vacancies.

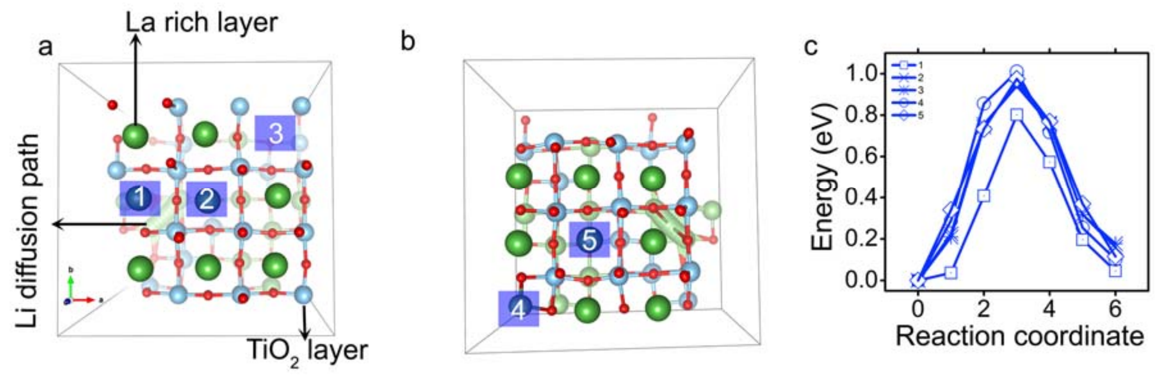

Supplementary Fig. 11 | Influence of LLTO A-site configurations on Li-ion migration in the defect layer. **a**,**b**, Five LLTO A-site configurations considered in Supplementary Note 1. **c**, Energy profiles of the same Li-ion migration pathway (pathway 2 in Fig. 5d) with the adjacent LLTO layer exhibiting five different A-site configurations indicated in **a** and **b**.

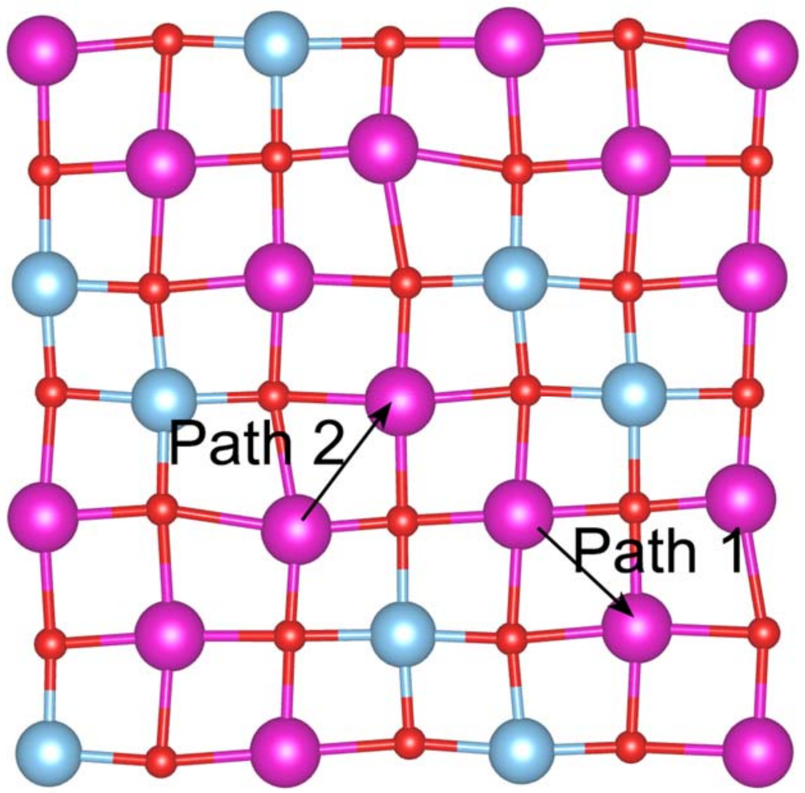

Supplementary Fig. 12 | Li-ion migration pathways in β-Li_2TiO_3. The (202) plane of β-Li_2TiO_3 has similar atomic configuration as the 2D defect (O: red, Li: purple, Ti: light blue). The Li-ion migration pathways selected for NEB calculations are indicated by arrows.

Supplementary Table 1. Estimated formation energies of the defect layer.

μ_{Li} (eV)	μ_O (eV)	ΔH_f (eV Å^{-2})
−1.75	0	0.249
−3.71	0	−0.009
−1.75	−3.91	−0.003
−3.71	−3.91	−0.262

Supplementary Note 1. Influence of LLTO A-site configurations on Li-ion migration in the defect layer

We analyzed the migration energy profiles of pathway 2 in Fig. 5d when the adjacent LLTO layer was showing five different local A-site configurations (Supplementary Fig. 11). Although the distribution of cations and vacancies in the neighboring LLTO layers varied significantly among these five configurations, the energy profile and energy barrier of Li-ion migration in the same pathway were found largely unchanged. This result suggests that the local A-site configuration of the adjacent LLTO layers exhibited negligible influence on the Li-ion migration within the 2D defect.

Supplementary Note 2. “Intrinsic conductivity” and “contributed conductivity” of SALTs

In the main text, the Li-ion migration within the defect layer was estimated to be slower than 2.43×10^{-12} S cm^{-1}. It should be emphasized that this conductivity value only describes how fast the Li ions were migrating within the specific atomic layer of the defect; its physical meaning is fundamentally different from that of the “SALT conductivity” determined by fitting the Nyquist plot. The conductivity obtained from the Nyquist plot actually reflects the contribution of all the defects within the LLTO pellet to the total conductivity, and thus is dependent on the population of the defects (this conductivity is referred to as “contributed conductivity” below). In contrast, the conductivity estimated below 2.43×10^{-12} S cm^{-1} here indicates the Li-ion diffusivity within the defect layer; it was derived purely from the atomic configuration of the 2D defect, and has nothing to do with the population of these defects in the material (this conductivity is referred to as “intrinsic conductivity” below).

In the present work, although the “intrinsic conductivity” of the defect can be estimated to be lower than 2.43×10^{-12} S cm^{-1}, the “contributed conductivity” cannot be straightforwardly determined. As shown in Supplementary Fig. 2, the SALTs did not induce an individual semicircle in the Nyquist plot. Therefore, the low-frequency semicircle, which was believed to arise from grain boundaries alone before the discovery of SALTs[1,2], may in fact be induced by the grain

boundaries and SALTs together. Because of this complication, it is very difficult (if possible) to reliably separate the "contributed conductivity" of SALTs from that of grain-boundaries in the Nyquist plot[3-5]. Therefore, as emphasized in the main text, the present study cannot conclude whether SALTs or grain boundaries are more severely degrading the total conductivity.

Supplementary Note 3. Formation energy of the 2D defect

The formation energy of the 2D defect is evaluated as follows. The defect slab consisted of 64 formula unit (f.u.) of LLTO ($Li_{0.3125}La_{0.5625}TiO_3$) and 20 f.u. of Li_2TiO_3 compensated with 36 Li atoms and 18 oxygen atoms. The formation energy can be evaluated as

$$\Delta H_\mathrm{f} = \frac{1}{2A}\left[E_\mathrm{LLTO}^\mathrm{slab} - 64E_\mathrm{LLTO}^\mathrm{bulk} - 20E_\mathrm{Li2TiO3}^\mathrm{bulk} + 18E_\mathrm{Li2O}^\mathrm{bulk}\right) \;\ldots\ldots(1)$$

where A is the area of the defect layer in the supercell used and 2 accounts for the presence of two defect layers in the slab. $E_\mathrm{LLTO}^\mathrm{slab}$ is the total DFT energy of the supercell with defect layers. $E_\mathrm{LLTO}^\mathrm{bulk}$, $E_\mathrm{Li2TiO3}^\mathrm{bulk}$, and $E_\mathrm{Li2O}^\mathrm{bulk}$ are the DFT energies of LLTO, Li_2TiO_3, and Li_2O, respectively. Using equation (1) and the DFT energies of all these structures, the formation energy of the defect layer can be estimated as ΔH_f = 0.113 eV Å^{-2}.

We further evaluated the formation energy of the defect layer under different external conditions during materials synthesis and processing, which affect the chemical potentials of lithium (μ_Li) and oxygen (μ_O)[6,7]. With these factors considered, the formation energy of the defect layer can be estimated as

$$\Delta H_\mathrm{f} = \frac{1}{2A}\left[\,E_\mathrm{LLTO}^\mathrm{slab} - 64E_\mathrm{LLTO}^\mathrm{bulk} - 20E_\mathrm{Li2TiO3}^\mathrm{bulk} + 36\,\mu_\mathrm{Li} + 18\,\mu_\mathrm{O}\right) \;\ldots\ldots(2)$$

Using the Materials Project[8], we obtained the stability ranges of μ_Li and μ_O (referenced to the elementary state as 0 eV in the Materials Project) for LLTO as -3.71 eV < μ_Li < -1.75 eV and $\mu_\mathrm{O} > -3.91$eV, respectively. The range of the formation energy ΔH_f can be evaluated within these ranges of chemical potentials. As shown in Supplementary

Table 1, the formation energy of the defect layer is negative at low values of μ_{Li} and/or μ_O. These results suggest that the formation of 2D defects is favored by lithium-poor and/or oxygen-poor conditions, which may very likely happen during the high-temperature sintering required by the synthesis of these materials[1].

Supplementary References

1. Bohnke, O. The fast lithium-ion conducting oxides $Li_{3x}La_{2/3-x}TiO_3$ from fundamentals to application. *Solid State Ionics* **179,** 9–15 (2008).
2. Inaguma, Y. *et al.* High ionic conductivity in lithium lanthanum titanate. *Solid State Commun.* **86,** 689–693 (1993).
3. Famprikis, T., Canepa, P., Dawson, J. A., Islam, M. S. & Masquelier, C. Fundamentals of inorganic solid-state electrolytes for batteries. *Nat. Mater.* **18,** 1278–1291 (2019).
4. Sångeland, C., Mindemark, J., Younesi, R. & Brandell, D. Probing the interfacial chemistry of solid-state lithium batteries. *Solid State Ionics* **343,** 115068 (2019).
5. Srivastav, S., Xu, C., Edström, K., Gustafsson, T. & Brandell, D. Modelling the morphological background to capacity fade in Si-based lithium-ion batteries. *Electrochim. Acta* **258,** 755–763 (2017).
6. Zhu, Y., He, X. & Mo, Y. First principles study on electrochemical and chemical stability of the solid electrolyte-electrode interfaces in all-solid-state Li-ion batteries. *J. Mater. Chem. A* **4,** 3253–3266 (2015).
7. He, X. & Mo, Y. Accelerated materials design of $Na_{0.5}Bi_{0.5}TiO_3$ oxygen ionic conductors based on first principles calculations. *Phys. Chem. Chem. Phys.* **17,** 18035–18044 (2015).
8. Jain, A. *et al.* A high-throughput infrastructure for density functional theory calculations. *Comp. Mater. Sci.* **50,** 2295–2310 (2011).
