# Supplementary material for: Single-atom-layer traps in a solid electrolyte for lithium batteries
Source: Nat Commun. 2020 Apr 14;11:1828. doi: 10.1038/s41467-020-15544-x (PMC7156726; doi:10.1038/s41467-020-15544-x)
Supplement: Supplementary file 2 — Description of Additional Supplementary Files [file 41467_2020_15544_MOESM2_ESM.pdf]

Description of Additional Supplementary Files

File Name: Supplementary Data 1

Description: CIF file describing the atomic model used for simulation
